# IgA vasculitis presenting as nephrotic syndrome following COVID-19 vaccination: a case report

**DOI:** 10.1186/s12882-022-03028-7

**Published:** 2022-12-15

**Authors:** Illeon Cho, Jwa-Kyung Kim, Sung Gyun Kim

**Affiliations:** Division of Nephrology, Department of Internal Medicine, Hallym University Sacred Heart Hospital, 22, Gwanpyeong-ro 170 beon-gil, Dongan-gu, Gyeonggi-do 14068 Anyang, Republic of Korea

**Keywords:** COVID-19 vaccine, Messenger RNA, IgA vasculitis, Case report, Nephrotic syndrome

## Abstract

**Background:**

Following the strong recommendation for coronavirus disease 2019 (COVID‑19) vaccination, many patients with medical comorbidities are being immunized. However, the safety of vaccination in patients with autoimmune diseases has not been well established. We report a new case of biopsy-proven IgA vasculitis with nephritis presenting as a nephrotic syndrome after mRNA COVID-19 vaccination in a patient with a history of leukocytoclastic vasculitis.

**Case presentation:**

A 76-year-old man with a history of cutaneous leukocytoclastic vasculitis presented with purpura in both lower limbs, followed by nephrotic syndrome after the second dose of BNT162b2 mRNA COVID-19 vaccination. Skin and renal biopsy revealed IgA vasculitis with nephritis. The patient’s past medical history of leukocytoclastic vasculitis and features of chronicity in renal pathology suggest an acute exacerbation of preexisting IgA vasculitis after COVID-19 vaccination. After the steroid and renin-angiotensin system inhibitor use, purpura and acute kidney injury recovered within a month. Subnephrotic proteinuria with microscopic hematuria remained upon follow-up.

**Conclusion:**

Physicians should keep in mind the potential (re)activation of IgA vasculitis following mRNA COVID-19 vaccines. It is important to closely monitor COVID-19 vaccinated patients, particularly those with autoimmune diseases.

## Background

The global COVID-19 vaccine campaign has been actively underway to overcome the COVID-19 pandemic. mRNA COVID-19 vaccines deliver lipid nanoparticle-encapsulated mRNA, which encodes the severe acute respiratory syndrome coronavirus 2 (SARS-CoV-2) spike protein and triggers robust innate and adaptive immune responses [1–3]. The efficacy and safety of BNT162b2 and mRNA-1273 have been demonstrated in clinical trials and real-world studies [4–7]. However, the safety of vaccination in patients with autoimmune diseases has not been well established.

Recently, de novo or relapsing glomerulonephritis (GN) following mRNA COVID-19 vaccines has been reported. IgA nephropathy was the most common GN among them, where most cases presented with gross hematuria shortly after the first or second dose of vaccination [8–10].

There is no diagnostic tool to prove the causality between GN and mRNA COVID-19 vaccines. However, the consistency of case reports may be related to its pathogenesis and will help to make a hypothesis to prove the causality, which will also help manage kidney disease properly. Herein, we report a new case of biopsy-proven IgA vasculitis with nephritis presenting as a nephrotic syndrome following the BNT162b2 mRNA COVID-19 vaccine in a patient with a history of cutaneous leukocytoclastic vasculitis.

## Case presentation

**Fig. 1:**
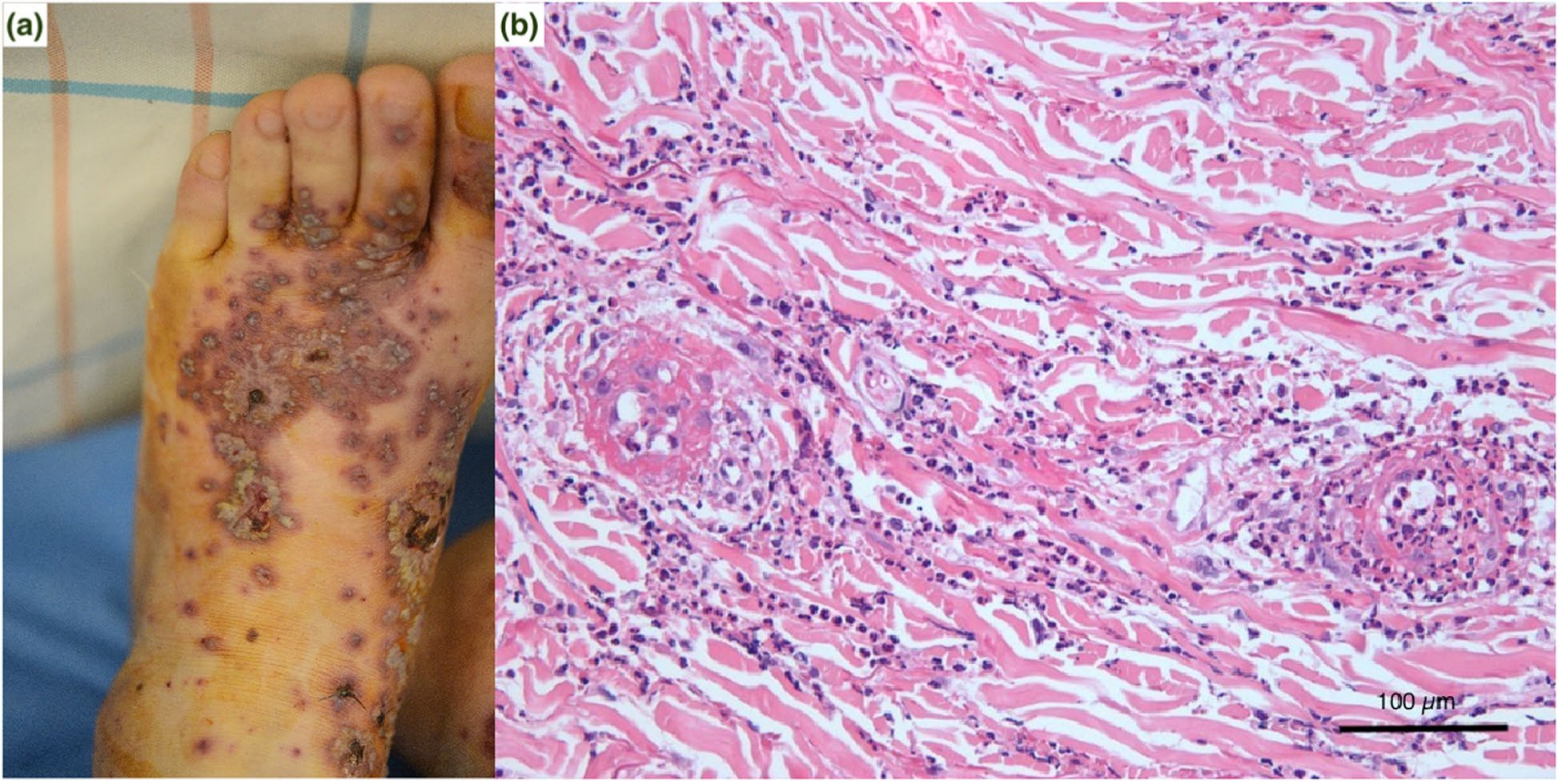
Dermatologic examination and skin biopsy findings. **a** Hemorrhagic papulovesicular lesions distributed on bilateral lower legs; **b** Skin biopsy showing perivascular inflammatory infiltration compatible with leukocytoclastic vasculitis (hematoxylin and eosin, original magnification x200)

The patient is a 76-year-old man with a history of leukocytoclastic vasculitis treated with immunosuppression therapy ten years ago and has been in remission with no medication. At that time, he presented with palpable purpuric rash on both lower limbs and had no other systemic signs of vasculitis such as abdominal pain, arthralgia, proteinuria, or decreased renal function. Skin biopsy revealed leukocytoclastic vasculitis, even though immunofluorescence staining was not performed. He was treated with oral prednisolone (20 mg per day) for two weeks, followed by oral azathioprine (25 mg per day) for six months since his skin lesions were severe and progressive. He visited the dermatology outpatient clinic with a sudden-onset purpuric rash on both lower extremities. Ten days prior to his visit, he received the second dose of the BNT162b2 mRNA COVID-19 vaccine (BioNTech-Pfizer). He had no history of COVID-19 infection or other recent respiratory, urinary or gastrointestinal disease and denied any symptoms between the first dose and the second dose of the mRNA vaccine. He was afebrile, and other vital signs were unremarkable. On physical examination, hemorrhagic papulovesicular lesions distributed on both lower legs were observed (Fig. 1a). The serum creatinine level was 1.06 mg/dL (eGFR 60 mL/min/1.73 m²), and urinalysis was not performed at that time. Skin biopsy was performed and revealed leukocytoclastic vasculitis (Fig. 1b). Oral prednisolone 20 mg per day for two weeks was prescribed, and the skin lesions began to be gradually relieved.

However, on day 30 after vaccination (second dose), diffuse abdominal pain and diarrhea were reported. On day 45 after vaccination (second dose), he was referred to the Department of Nephrology for worsening peripheral edema and was hospitalized. Vital signs were unremarkable except for a blood pressure of 140/80 mmHg. Physical examination revealed 3 + pretibial pitting edema with multiple purpuric rashes on both lower limbs.

Serum creatinine increased from 1.06 to 1.42 mg/dL (eGFR decreased from 60 to 48 mL/min/1.73 m²), and serum albumin decreased from 3.8 to 2.7 g/dl. Nephrotic-range proteinuria was observed; urinalysis showed 4 + protein, 10–19 red blood cells (RBCs)/high power field (HPF), and 10–19 white blood cells (WBCs)/HPF, and the spot urine protein/creatinine ratio (UPCR) was 9.01 mg/mg. Tests for hepatitis B surface antigen and antibodies to hepatitis C virus were negative; C3 and C4 were within normal reference ranges; tests for anti-nuclear antibodies, anti-extractable nuclear antigen antibodies, anti-neutrophil cytoplasm antibodies, and anti-glomerular basement membrane antibodies were negative. The polymerase chain reaction test for COVID-19 (nasopharyngeal swab) was negative. Renal sonography revealed normal-sized kidneys with normal parenchymal echogenicity. Table 1 summarizes clinical information.

**Table 1:** Patient demographics and clinical information

**Clinical presentation**		
Age, yr/race/sex		76/Korean/Male
Medical history		Leukocytoclastic vasculitis, 10 years ago
Vaccine name		BNT162b2 (BioNTech-Pfizer)
Date of vaccination		
First dose		April, 24, 2021
Second dose		May, 14, 2021
Timing of purpura occurrence after the second dose		10 days
Timing of nephrotic syndrome occurrence after the second dose		45 days
Other associated symptoms		Abdominal pain, diarrhea
**Laboratory datas (after the second dose of vaccine)**		
On day 10	Serum creatinine, mg/dl	1.06
	Serum albumin, g/dl	3.8
	UPCR, mg/mg	NA
	RBC, cells/μl	NA
On day 45	Serum creatinine, mg/dl	1.42
	Serum albumin, g/dl	2.7
	UPCR, mg/mg	9.01
	RBC, cells/μl	20–29
On day 60	Serum creatinine, mg/dl	1.04
	Serum albumin, g/dl	3.0
	UPCR, mg/mg	8.07
	RBC, cells/μl	20–29
On day 120	Serum creatinine, mg/dl	1.03
	Serum albumin, g/dl	3.5
	UPCR, mg/mg	1.97
	RBC, cells/μl	10–19
**Histopathology**		
Glomeruli		27 glomeruli; 4 (15%) global sclerosis, 10 (37%) cellular or fibrocellular crescents, 2 (7%) segmental sclerosis
Tubules and interstitium		Focal severe tubule atrophy and fibrosis in interstitium (< 10% of cortical area)
Vessels		Mild arteriolosclerosis
Immunofluorescence		Dominant IgA staining
Electron microscopy		Moderate amounts of mesangial deposits; normal thickness of GBM; focal moderate effacement of epithelial cell foot processes
**Treatment**		I.V. Methylprednisolone 250 mg for 3 days followed by oral prednisolone; olmesartan 40 mg

*UPCR* urine protein-to-creatinine ratio, *RBC* urine red blood cell count, *GBM* glomerular basement membrane, *NA* not applied

**Fig. 2:**
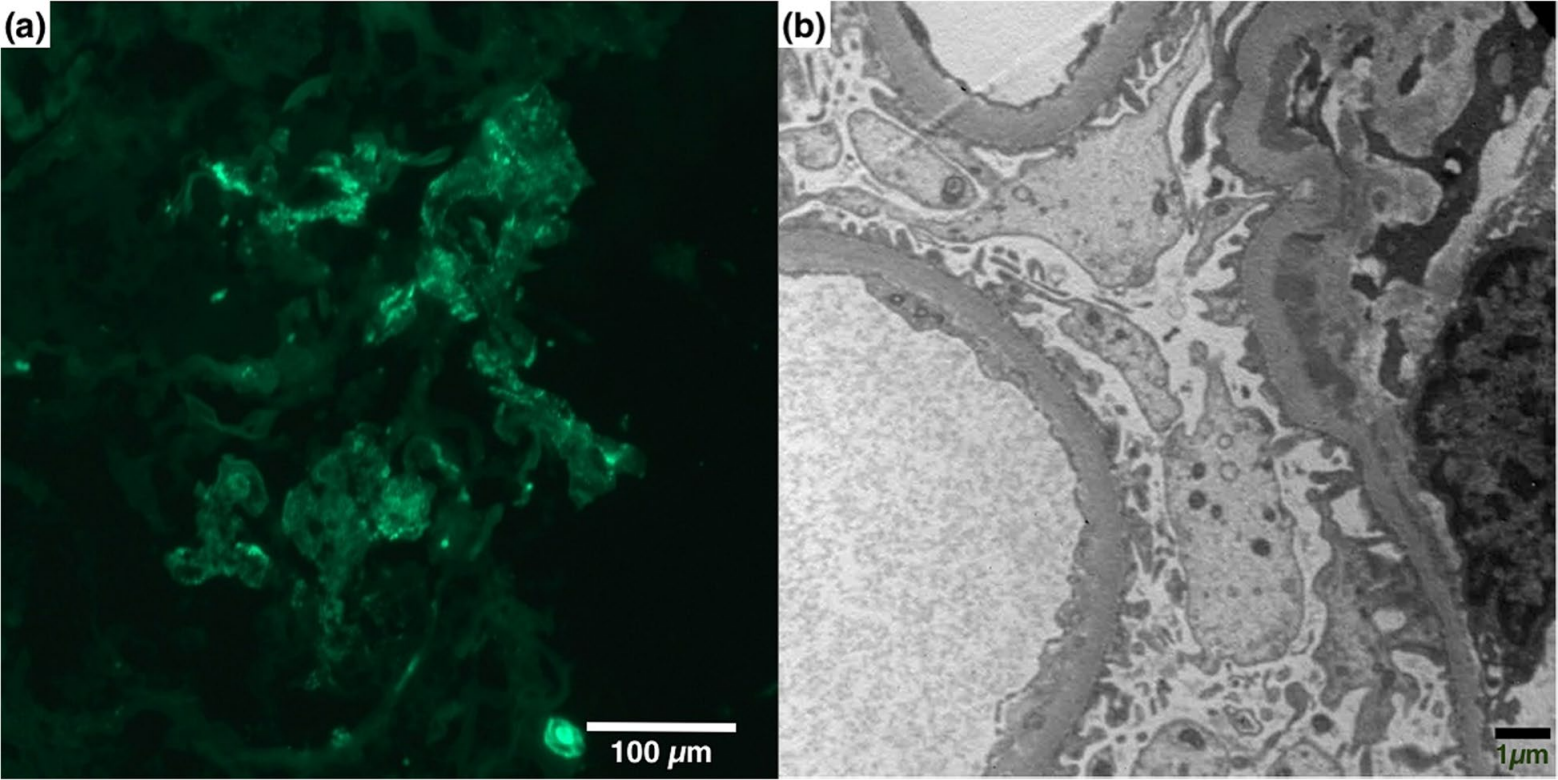
Renal biopsy findings. **a** Immunofluorescence images captured on an Axioplan2 microscope (Zeiss, Gottingen, Germany) equipped with HXP 120 V lamp, a 12 bit Axiocam CCD camera (Zeiss), a motorized object desk and filter changer were controlled by SFM software (3DHistech Ltd, Budapest, Hungary) shows predominant mesangial IgA staining with partial peripheral staining (original magnification x400); **b** Electron microscopy (Hitachi H-7100, Tokyo, Japan) shows moderate amounts of mesangial deposits and focal moderate effacement of epithelial cell foot processes (original magnification x8000)

A kidney biopsy was performed 45 days after the second dose of vaccine. In total, 27 glomeruli were identified, 4 of which showed global sclerosis, 10 glomeruli showed cellular or fibrocellular crescents, and 2 glomeruli showed segmental sclerosis. Focal severe tubular atrophy with infiltration of mononuclear cells and fibrosis in the interstitium were observed in approximately less than 10% of the cortical area. Immunofluorescence microscopy showed predominant mesangial IgA staining with partial peripheral staining, which is compatible with IgA nephritis (Fig. 2a). Electron microscopy revealed moderate amounts of mesangial deposits with podocytes, which showed focal foot process effacement (Fig. 2b).

With the diagnosis of IgA vasculitis with nephritis presenting as nephrotic syndrome, intravenous methylprednisolone 250 mg was given for three consecutive days, followed by oral prednisolone 50 mg per day with olmesartan 40 mg daily. One week later, serum creatinine decreased to 1.04 mg/dL, the UPCR was 8.07 mg/mg, and he was discharged with continued prednisolone and olmesartan. At the 2-month follow-up, the rash and edema were much improved. Serum creatinine level was 1.03 mg/dL, and the UPCR was reduced to 1.97 mg/mg. After that, prednisolone was tapered gradually over four months. Six months after the start of the patient’s treatment, his serum creatinine level was 1.0 mg/dL, the UPCR had decreased to 1 mg/mg, and the microscopic hematuria remained. The drugs were well tolerated, with no significant adverse effects.

## Discussion and conclusions

Since the COVID-19 pandemic, de novo or relapsing glomerulonephritis following mRNA COVID-19 vaccination has been reported. IgA nephropathy is the most common GN after receiving the mRNA COVID-19 vaccine, followed by minimal change disease [8–10]. In the literature, 47 cases of IgA nephropathy following mRNA COVID-19 vaccination have been reported (30 de novo and 17 flare-ups) thus far [3, 9–40]. Gross hematuria within 1–2 days after vaccination was the most common initial presentation, followed by acute kidney injury. Symptoms tended to appear after the second dose of the vaccine instead of after the first dose of the vaccine. Most cases spontaneously resolved with supportive care, such as renin-angiotensin system inhibitors, but in a few cases, immunosuppressive therapy was applied.

Compared to previous cases, our patient initially presented with palpable purpura on the lower limbs followed by nephrotic syndrome, not gross hematuria, and it occurred relatively late after the second dose of vaccine. In addition, skin and renal biopsy revealed IgA vasculitis with nephritis. Although we cannot rule out the possibility that IgA vasculitis coincidently occurred after the COVID-19 vaccination in our case, the past medical history of leukocytoclastic vasculitis and the features of chronicity in renal pathology suggest the possibility that the COVID-19 vaccination triggered an acute exacerbation of preexisting IgA vasculitis. However, it is impossible to confirm whether IgA deposits were present in the kidney tissue before the COVID-19 vaccination since the patient had never performed a renal biopsy before the COVID-19 vaccination.

Nephrotic syndrome is a rare presentation of IgA nephropathy [41]. Indeed, to our knowledge, only three previous cases [22, 36] described biopsy-proven IgA vasculitis with severe glomerulonephritis after Pfizer-BioNTech COVID-19 vaccination, which presented with nephrotic syndrome. Histopathologically, two cases [36] were IgA nephropathy with glomerular capillary IgA deposition, a rare subtype of IgA nephropathy, and one case [22] showed necrotizing cellular crescent formation. Despite intensive initial immunosuppressive treatment, its severe glomerulonephritis took a long time to recover.

The safety and efficacy of immunosuppressive drugs in treating adult-onset IgA vasculitis with renal involvement are not well established. Almost all data come from studies carried out in children or patients with IgA nephropathy or IgA-crescentic glomerulonephritis [42]. Usually, immunosuppressive therapy is not recommended as the initial treatment of choice for patients with IgA nephropathy in adults. It is recommended only for those at high risk of progressive chronic kidney disease despite maximal supportive care or some special situations [43].

In our case, we treated the patient with glucocorticoids immediately since he presented with nephrotic syndrome. Indeed, recent KDIGO guidelines recommend using glucocorticoids in some patients with IgA nephropathy presented with nephrotic syndrome (including edema, both hypoalbuminemia and nephrotic-range proteinuria > 3.5 g/day) as this condition resembles podocytopathy such as minimal change disease [43]. In our case, AKI and the nephrotic syndrome were recovered after one week and two months of high-dose glucocorticoid treatment, respectively. Moreover, complete remission has been sustained with a low dose of glucocorticoids for the subsequent four months.

The causality between mRNA COVID-19 vaccines and IgA vasculitis is unclear. However, several pieces of evidence support possible mechanisms of how mRNA COVID-19 vaccines may induce autoimmunity. Evidence includes hypotheses related to the antigen-specific trigger by molecular mimicry between SARS-CoV-2 proteins and human tissue and to the antigen-nonspecific trigger by dysregulation of cytokine [1, 2, 44, 45]. In particular, IgA vasculitis with nephritis and IgA nephropathy share a four-hit hypothesis for pathogenesis; in this process, various cytokines are associated with autoimmunity [46]. Previous case reports have suggested that upper respiratory tract infection by SARS-CoV-2 can trigger IgA vasculitis [47–50]. IgA vasculitis following mRNA COVID-19 vaccine may share some occurrence mechanisms with IgA vasculitis in the COVID-19 infection setting. Further case reports and studies are required to understand the pathogenesis.

It is reasonable to advise patients to receive COVID-19 vaccination despite having prior or current IgA vasculitis, considering that most previous cases already reported were with a favorable prognosis. However, they need to be followed up more closely, especially if they are in remission and without any treatment.

## Data Availability

The data and materials are available from the corresponding author, upon reasonable request.

## Abbreviations

COVID-19: Coronavirus disease 2019
SARS-CoV-2: Severe acute respiratory syndrome coronavirus 2
GN: Glomerulonephritis
eGFR: Estimated glomerular filtration
UPCR: Urine protein/creatinine ratio
RBCs: Red blood cells
WBCs: White blood cells
HPF: High power field
